# Reviewer acknowledgements

**DOI:** 10.1186/1472-6815-13-3

**Published:** 2013-02-21

**Authors:** Christopher Foote

**Affiliations:** 1 BMC Series, BioMed Central, 236 Grays Inn Road, WC1X 8HB, London, UK

## Abstract

**Contributing reviewers:**

The editors of BMC Ear, Nose and Throat Disorders would like to thank all our reviewers who have contributed to the journal in volume 12 (2012).

## 

Zubair Ahmed

USA

Mazita Ami

Malaysia

Daiya Asaka

Japan

Swee Aw

Australia

Patrick Boyle

UK

Gabriella Cadoni

Italy

Ning-Chia Chang

Taiwan

Jean-Francois Chenot

Germany

Helen Cohen

USA

Jacquelynne Corey

USA

Erhan Demirhan

Turkey

Richard Dowell

Australia

Eva Ekvall Hansson

Sweden

Bing-Jian Feng

USA

Peter Flipsen Jr

USA

Robert Folmer

USA

Balwant Singh Gendeh

Malaysia

Bee See Goh

Malaysia

Quinton Gopen

USA

Robin Green

South Africa

Catherine Ann Haighton

UK

Atsushi Harimaya

Japan

Hiroshi Hidaka

Japan

Susan Hillier

Australia

Niels Henrik Ingvar Hjollund

Denmark

Kathrine Jauregui-Renaud

Mexico

Tino Just

Germany

John Krouse

USA

Berthold Langguth

Germany

Faramarz Memari

Iran

Regina Celia Mingroni-Netto

Brazil

Kensei Naito

Japan

Jerry Polesel

Italy

Mandana Rafeey

Iran

Pascal Senn

Switzerland

Sverre Steinsvaring

Norway

Boris A. Stuck

Germany

Krister Tano

Sweden

Ligy Thomas

UK

Yasue Uchida

Japan

Martin Vestergaard

UK

Magnus von Unge

Norway

Lesley Voss

New Zealand

Cornelia Weise

Sweden

Jiunn-Liang Wu

Taiwan

Hideo Yonezawa

Japan

## Pre-publication history

The pre-publication history for this paper can be accessed here:

http://www.biomedcentral.com/1472-6815/13/3/prepub

